# Physical Activity and Psychological Wellbeing Among Healthcare Students During the COVID-19 Pandemic

**DOI:** 10.7759/cureus.55577

**Published:** 2024-03-05

**Authors:** Shahinaz N Sembawa, Abdulrahman S Jabr, Asrab A Banjar, Haneen S Alkuhayli, Modhi S Alotibi, Reem B AlHawsawi, Yara A Nasif, Arwa U AlSaggaf

**Affiliations:** 1 Division of Dental Public Health, Department of Preventive Dentistry, College of Dental Medicine, Umm Al-Qura University, Makkah, SAU; 2 College of Public Health and Health Informatics, Umm Al-Qura University, Makkah, SAU; 3 College of Applied Medical Sciences, Umm Al-Qura University, Makkah, SAU; 4 Oncology, King Faisal Specialist Hospital and Research Centre, Riyadh, SAU; 5 College of Nursing, Umm Al-Qura University, Makkah, SAU; 6 Division of Prosthodontics, Department of Oral and Maxillofacial Surgery and Diagnostic Science, College of Dental Medicine, Umm Al-Qura University, Makkah, SAU

**Keywords:** covid-19, healthcare, health-related students, psychological wellbeing, physical activity

## Abstract

Introduction: Regular physical activity (PA) contributes to physical and mental wellbeing. Due to coronavirus disease 2019 (COVID-19)-related lockdowns in 2020, regular PA had been adjusted. Furthermore, students became accustomed to studying at home. Students in the healthcare field, in particular, have a better understanding of the link between PA and mental health. This study aimed to assess the association between psychological wellbeing and PA among healthcare students at one public university in Saudi Arabia during the COVID-19 pandemic period.

Methods: This was a cross-sectional study conducted between November 2021 and February 2022. Healthcare students at one public university in Saudi Arabia were invited to participate in an online survey, consisting of three sections. The first section contained questions about demographic data. The second section was the International Physical Activity-Short Form (IPAQ-SF), which is composed of seven questions designed to assess the level of PA. The third part is The Center for Epidemiological Studies Depression Scale (CES-D), which is a self-reported questionnaire comprising 20 questions that assessed depressive symptoms on a four-point scale. Descriptive statistics, Chi-square test, Mann-Whitney U test, and Kruskal Wallis test were used to analyze the collected data using IBM SPSS Statistics for Windows, Version 28.0 (Released 2021; IBM Corp., Armonk, New York, United States).

Results: The total responses received were 197 (response rate=60%). The majority of students in this sample were performing inadequate PA and reported depressive symptoms. No association was found between the amount of PA practiced and psychological wellbeing. There was a significant difference between specialties in relation to PA.

Conclusion: The majority of students in this sample were performing inadequate PA and reported depressive symptoms during the COVID-19 lockdown. The importance of PA should be promoted among healthcare students.

## Introduction

The WHO defines physical activity (PA) as “any body movement produced by skeletal muscles that require energetic expenditure. PA means all movements, including recreational movements, for transportation to and from places, or as part of a person's work” [1]. The importance of PA has been proven in different aspects of human health and wellbeing. Studies suggested a linear relationship between human beings’ health and the volume of PA [2]. Performing regular PA contributes to the prevention and management of non-communicable chronic diseases such as cardiovascular diseases and diabetes mellitus [2]. It's important to maintain physical fitness, which can contribute to maintaining a healthy weight, building and maintaining healthy bone density, muscle strength, and joint mobility, promoting physiological and psychological wellbeing, strengthening the immune system, reducing risk factors of cardiovascular disease, and helping to prevent death from cancer [2-4]. In fact, the dismissal of physical inactivity would reduce 6-10% of chronic heart disease, type 2 diabetes, and breast and colon cancers, and increase life expectancy [4]. The WHO recommends “150 minutes of moderate-intensity or 75 minutes of vigorous-intensity PA per week, or a mixture of both” [1].

Evidence shows that being physically active has a relation to higher quality of life [3,5,6] which indicates good physical as well as mental wellbeing. For mental wellbeing, long-term PA increases the expression of brain growth factors and causes the release of endorphins in the brain, which may lead to a sense of calm and improved mood after exercise [7, 8]. Also, it has a significant impact on reducing the risk of clinical depression, and the ability to influence acute anxiety feelings or relatively stable anxiety traits [7,8].

Coronavirus disease 2019 (COVID-19), which began in China at Wuhan [9-11] was declared a pandemic by the WHO on March 11, 2020 [11]. Spreading all over the world, which was a significant threat to people's health, the pandemic resulted in the implementation of global restrictive measures to reduce social contact and restricted movement to reduce the spread of the virus, that placed unprecedented restrictions on people’s physical activities and routines. Quarantine [11] online learning, and closure of gyms and other recreational facilities as a restrictive measure resulted in a reduction in students’ learning and development levels [9]. Moreover, the COVID-19 crisis affected people's psychological wellbeing [12].

The putative role of PA in mental health promotion, prevention of mental health problems, and academic achievement among the young has been increasingly debated [13,14]. Some studies showed a positive relationship between PA and academic achievement [15-17], while other studies showed weak and inconclusive associations [5,18,19].

Physical inactivity and sedentary behaviour are related to poor physical and mental health [4]. A study showed that lower levels of PA were linked to higher depressive symptoms among university students and since students are vulnerable to psychological distress, it’s important to maintain proper PA [8]. Physical self-esteem, sleep, and cognitive function can be improved by PA, which is also important for social interaction and wellbeing [5].

In general, PA positively impacts mental health providing a better quality of life. Furthermore, it is presumed that medical students are conscious of the importance of healthy living and keeping an active lifestyle [7]. There is a need to better understand factors that determine whether our future healthcare providers choose to be active or sedentary, as their own behaviour was found to affect their PA counselling and promotion [7]. Therefore, action should be taken to increase the likelihood that these individuals adopt healthy lifestyles before they enter the public health workforce [3, 8].

Therefore, this study focused on the practice of PA by healthcare undergraduate students during the COVID-19 pandemic. The aim was to determine the association between psychological wellbeing and PA among health-related students at one public university in Saudi Arabia during the COVID-19 period.

## Materials and methods

This was a cross-sectional online survey to determine the relationship between psychological well-being and PA among healthcare students during the COVID-19 period. This study was conducted at Umm Al-Qura University in Makkah, Saudi Arabia, from November 2021 to February 2022. The study was approved by the Biomedical Research Ethics Committee at Umm Al-Qura University (approval number: HAPO-02-K-012-2021-11-829).

The questionnaire

The questionnaire was developed online using Microsoft Forms (Microsoft Corporation, Redmond, Washington, United States) and was composed of three parts. The first part included questions about sociodemographic data (age, gender, city, specialty, and academic year). The second part was the International Physical Activity-Short Form questionnaire (IPAQ-SF) which consists of seven questions designed to measure the level of PA [20]. Participants were asked to record their PA during the lockdown period. The third section consisted of the Center for Epidemiologic Studies Depression Scale (CES-D) which is a self-reported questionnaire comprising 20 questions that assess depressive symptoms on a four-point scale in the general population [21]. Participants were asked to record how they felt during the lockdown period.

Distribution

Questionnaires were sent to class leaders of all years of both male and female sections of health-related colleges in a public university, and they were asked to distribute the questionnaire among their colleagues. Reminders were sent to class leaders to remind their colleagues to fill out the questionnaire. Non-healthcare students were excluded from this study.

Statistical analysis

A sample size of 328 was calculated for this study using Epitools online calculator (Ausvet, Fremantle, Australia) with 80% power, 95% confidence interval (CI), and 5% margin of error. The collected data was analyzed using IBM SPSS Statistics for Windows, Version 28.0 (Released 2021; IBM Corp., Armonk, New York, United States). Descriptive statistics, Chi-square test, Kruskal Wallis test, and Mann-Whitney U test were used to explore associations between psychological well-being and PA. The significance level was set at p-value <0.05.

## Results

In total, 197 healthcare students responded to the survey (response rate = 60%) with an age range of 18-25 (mean=21.44) years, of which 69 were males (35%) and 128 were females (65%). Of the total sample, the majority of the responses were from Applied Medical Sciences students (28.9%), followed by Public Health and Health Informatics (22.8%), then medical students (14.2%), dental students (13.7%), nursing students (10.7%), and pharmacology students (9.6%) (Table 1).

**Table 1:** Participant characteristics

Characteristics	Frequency	Percentage
Gender		
Female	128	65%
Male	69	35%
Student Specialties		
Applied Medical Sciences	57	28.9%
Public Health and Health Informatics	45	22.8%
Medicine	28	14.2%
Dentistry	27	13.7%
Nursing	21	10.7%
Pharmacy	19	9.6%
Academic Year		
2nd Year	42	21.3%
3rd Year	52	26.4%
4th Year	52	26.4%
5th Year	16	8.1%
6th Year	10	5.1%
Internship	25	12.7%
Physical activity	Median±SD	
Moderate intensity exercise (min/week)	0±153.8	
Vigorous intensity exercise (min/week)	0±127.2	
Walking days (days/week)	4±2.5	
Sitting time (h/day)	0±5.5	

PA among healthcare students during the COVID-19 period

The majority of the students in this sample were performing inadequate vigorous activity (78.7%) (median=0±127.2) and inadequate moderate PA (85.8%) (median= 0±153.8). A total of 54 students (27.4%) performed adequate vigorous or moderate activity or a combination of both. Those who performed adequate PA reported (median= 358.5 minutes/week ±265.1) compared to those who performed inadequate PA (median=0±38.4) (Table 2).

**Table 2:** Performance of physical activity by gender, college, and school year

Characteristics	Vigorous activity		Moderate activity	
	Inadequate, n (%)	Adequate, n (%)	Inadequate, n (%)	Adequate, n (%)
Gender				
Male	54 (27.4%)	15 (7.6%)	56 (28.4%)	13 (6.6%)
Female	101 (51.3%)	27 (13.7%)	113 (57.4%)	15 (7.6%)
Student Specialties				
Applied Medical Sciences	45 (22.8 %)	12 (6.1%)	50 (25.4 %)	7 (3.6%)
Nursing	21 (10.7%)	0 (0%)	20 (10.2%)	1 (0.5%)
Public Health and Health Informatics	30 (15.2%)	15 (7.6%)	35 (17.8%)	10 (5.1%)
Dentistry	21 (10.7%)	6 (3%)	19 (9.6%)	8 (4.1%)
Medicine	24 (12.2%)	4 (2%)	28 (14.2%)	0 (0%)
Pharmacology	14 (7.1%)	5(2.5 %)	17 ( 8.6%)	2 (1 %)
Total	155 (78.7 %)	42(21.3%)	169(85.8%)	28 (14.2 %)
Academic year				
2^nd^ Year	33 (16.8%)	9 (4.6%)	39 (19.8%)	3 (1.5%)
3^rd^ Year	42 (21.3%)	10(5.1%)	43 (21.8%)	9 (4.6%)
4^th^ Year	41 (20.8%)	11 (5.6%)	42 (21.3%)	10 (5.1%)
5^th^ Year	12 (6.1%)	4 (2%)	13 (6.6%)	3 (1.5%)
6^th^ Year	7 (3.6%)	3 (1.5%)	8 (4.1%)	2 (1%)
Internship	20 (10.2%)	5 (2.5%)	24 (12.2%)	1 (0.5%)
Total	155 (78.7%)	42 (21.3%)	169 (85.8%)	28 (14.2%)

Table 2 shows the performance of PA by gender, specialty, and school year. The majority of students, irrespective of gender, performed inadequate vigorous and moderate activity; however, the proportion of females performing adequate vigorous and moderate activities was higher (13.7% and 7.7 %, respectively) compared to males (7.6% and 6.6%, respectively).

Students of Public Health and Health Informatics reported the highest performance of adequate vigorous and moderate activities among the study sample (7.6 % and 5.1%, respectively). On the other hand, nursing students reported no adequate vigorous activity, while medical students reported no adequate moderate activity. Moreover, students of Applied Medical Sciences reported the highest percentage of inadequate vigorous and moderate activities among the study sample (22.8% and 25.4%, respectively).

Fourth-year students reported the highest performance of adequate vigorous and moderate activity (5.6% and 5.1%, respectively), while third-year students reported the highest inadequate vigorous and moderate activity (21.3% and 21.8%, respectively). There was a significant difference between students according to specialties in the practice of vigorous (P=0.03) and moderate activities (P = 0.01).

Psychological wellbeing among healthcare students during the COVID-19 period

The results of psychological wellbeing showed that the majority of the respondents reported depressive symptoms )63.5%; mean=21.4 ±12.7). Significantly more females reported depressive symptoms compared to males (44.2% vs. 19.3%) (P=0.026) (Table 3). There was no significant association between physical activity and psychological wellbeing in this study sample (P=0.8).

**Table 3:** Psychological wellbeing among healthcare students during the COVID-19 period COVID-19: coronavirus disease 2019

Characteristics	Psychological status	
	No depressive symptoms, n (%)	Depressive symptoms, n (%)
Gender		
Male	31 (15.7%)	38 (19.3%)
Female	41 (20.8%)	87 (44.2%)
Student Specialties		
Applied Medical Sciences	19 (9.6%)	38 (19.3%)
Nursing	8 (4.1%)	13 (6.6%)
Public Health and Health Informatics	12 (6.1%)	33 (16.8%)
Dentistry	15 (7.6%)	12 (6.1%)
Medicine	11(5.6%)	17 (8.6%)
Pharmacology	7 (3.6%)	12 (6.1%)
Academic year		
2^nd^ Year	16 (8.1%)	26 (13.2%)
3^rd^ Year	15 (7.6%)	37 (18.8%)
4^th^ Year	20 (10.2.6%)	32 (16.2%)
5^th^ Year	5 (2.5%)	11 (5.6%)
6^th^ Year	6 (3%)	4 (2%)
Internship	10 (5.1%)	15 (7.6%)
Total	72 (36.5%)	125 (63.5%)

## Discussion

This study aimed to assess the association between psychological wellbeing and PA among healthcare students in a public university during the COVID-19 period. The results showed that the majority of students were practicing inadequate PA, and the majority reported depressive symptoms. Significantly, more females reported depressive symptoms than males. There was no significant association between the practice of PA and psychological well-being in this study sample.

According to recent studies from different countries, the COVID-19 crisis had psychological impacts on people [12,22-26]. In Saudi Arabia, it was found that 41.4% of the sample population exhibited moderate depression with 15.2% exhibiting severe or extreme depression [26]. A study from Kuwait reported that 59.6% of their sample population suffered from depression during the quarantine period [25]. These findings come in line with the findings of this study, as the majority of this study sample was at risk of depression. This is related to social isolation, the suspension of schools and universities, and the need for online education.

Psychological impacts were found greater on women, younger individuals, and individuals with lower levels of education [22-24,27]. According to a study in Saudi Arabia, females exhibited more severe, extreme, and moderate depression compared to males, who exhibited mild and borderline depression [26]. These findings are consistent with the findings of the current study, where more females reported depressive symptoms than males with a significant difference. This could be related to the psychological nature of females who can be more likely to develop anxiety and depression than males.

The majority of students in the current study were performing inadequate PA during the COVID-19 lockdown. Similar findings were reported among college students from different countries around the globe [23-25,28,29], and it comes in line with the findings of a Saudi study, that reported a rise in inactivity by 21% during the quarantine period compared to pre-quarantine [26]. However, other nations like Italy and Spain have reported increased levels of PA during the lockdown periods [9,26,30].

It is well-documented in the literature that a sedentary lifestyle imposes a risk factor for depression [31]. Several studies reported associations between the level of PA and depression [27,32]. Chinese college students who engaged in a high level of PA had lower anxiety than those who engaged in low levels of PA, while individuals who engaged in moderate and high levels of exercise had lower depression than those with a low level of PA [23,33]. In the United States, college students reported fewer minutes of PA and elevated depression levels during quarantine [34]. This could explain the findings of this study, as the majority of students were not practicing adequate PA, thus they were at risk of depression. However, no significant association was found in the current study between the amount of PA and depression, consistent with another study from Sweden [35].

The current study expands the literature on PA and mental health during the COVID-19 outbreak and points to the need to promote PA among healthcare students to maintain their physical and mental wellbeing. More efforts need to be directed towards increasing students’ engagement with PA in health colleges. 

The strength of this study is the use of validated questionnaires to assess PA and depression status. However, the findings of this study need to be interpreted in light of its limitations. The use of self-reported surveys may result in recall bias. Therefore, it would have been more accurate to measure PA using accelerometry, with an expert assessment of psychological status, which could be a future research recommendation. Furthermore, baseline data for PA and psychological wellbeing is not available to facilitate comparisons. Moreover, this is a cross-sectional study design, thus the findings cannot be generalized. Additionally, the low response rate and the small number of each of the subgroups may have prevented the detection of significant associations.

## Conclusions

The majority of healthcare students in this study sample were performing inadequate physical activity and reported depressive symptoms during the COVID-19 period. However, there was no association found between the amount of physical activity performed and psychological status. It is important to raise awareness of the importance of physical activity among healthcare students and the community.
